# Climate Change Awareness: Does It Affect the Fertility Desire?

**DOI:** 10.1111/phn.13568

**Published:** 2025-05-15

**Authors:** Özge Topsakal, Esra Çevik

**Affiliations:** ^1^ Department of Obstetrics and Gynecology Nursing Manisa Celal Bayar University Manisa Türkiye; ^2^ Department of Midwifery Balıkesir University Balıkesir Türkiye

**Keywords:** awareness, climate change, fertility, women's health

## Abstract

**Aim:**

The primary aim of the study was to explore the impact of women's climate change awareness on fertility desire, while the secondary aim was to explore the factors influencing women's fertility desire.

**Material and Methods:**

The study was performed descriptive and correlational type with 440 women without children between March and October 2023. The women's characteristics form and Fertility Desire Scale and Climate Change Awareness Scale were used for data collection.

**Results:**

The mean age of the women was 26.2 ± 4 years, with 55.2% aged 26 or younger; 54.3% held university degrees, and 93% had health insurance. Findings indicate a significant mean total score of 50.7 ± 7.6 on the Fertility Desire Scale, influenced by education, health insurance, income level, marital duration, and type of marriage. Women with higher education levels and those in couple‐initiated marriages reported lower fertility desire. The Climate Change Awareness Scale showed a mean score of 210.8 ± 23.2, with higher awareness among those over 26, employed, and with higher education. Multiple linear regression analysis revealed significant predictors of fertility desire, including marital duration and income level. Notably, climate change awareness negatively correlated with fertility desire, explaining 3.1% of the variance.

**Conclusions:**

These findings highlight the complex interplay between environmental concerns and reproductive intentions among women, indicating a need for further research in this area.

## 1 Introduction

In recent years, climate change has become a significant global concern, influencing not only environmental conditions but also demographic trends, such as fertility rates and population aging. Global population changes, including declining birth rates and an increasing aging population, are particularly concerning for both developed and developing countries. In Türkiye, fertility rates ranged between 2.1 and 2.3 from 2001 onwards; however, a decline began after 2014, dropping to 1.5 in 2023 (Yadigaroğlu 2023; Turkish Statistical Institute 2024). This decline, combined with the increasing aging population, has brought pronatalist policies to the agenda, highlighting the need to better understand factors influencing reproductive intentions.

While socioeconomic development, family planning programs, maternal and child health improvements, and women's education and empowerment have all contributed to declining fertility rates (Bongaarts 2020; United Nations 2024), climate change has recently emerged as a potential factor influencing reproductive decisions. Eco‐anxiety, defined as the concern individuals feel about the future due to climate change, is considered one of the psychological disorders of the new era (Schneider‐Mayerson and Leong 2020). The 2021 report of The Lancet Countdown study emphasizes that multiple factors related to climate change, such as temperature increases, rising food prices, malnutrition, and the rise in respiratory diseases, will negatively impact the health of future generations (Romanello et al. 2021). Literature suggests that some young people express concerns about the potential impacts of climate change on their current or future children (Arnocky et al. 2012; Thomas et al. 2018; Schneider‐Mayerson and Leong 2020; Helm et al. 2021). In a study found that 11% of participants were “concerned about climate change” (Miller 2018), while another study revealed that 14.3% stated that the “main reason” for not having children was climate change (Morning Consult 2020). Conversely, one study indicated that some individuals perceive having children as a form of security against environmental threats (Haq 2018).

A systematic analysis examined publications on climate change, mental health, and reproductive decisions, identifying themes related to the desire for parenthood, such as uncertainty about the future of an unborn child, environmentalist views focused on overpopulation and overconsumption, the need to support family livelihood, and environmental and political sentiments. It was emphasized that the studies largely covered countries in the Global North, and therefore, the research was limited in terms of geographic diversity (Dillarstone et al. 2023). This study was original in its exploration of the impact of climate change awareness on women's fertility desire, a topic that remained under‐researched at both the national and international levels.

Climate change and its associated economic burdens and environmental impacts, such as droughts, fires, and floods, have intensified in Türkiye, with projections suggesting that their intensity, frequency, and affected areas will continue to increase (Turkes et al. 2020; Yücel and Kurnaz 2021). Given the country's vulnerability and limited coping capacity as a developing nation, understanding the influence of climate change on reproductive choices is critical. Therefore, the aim of this study was to explore the factors influencing women's fertility desire and how climate change awareness affects it.

The research questions include the following:

1. Is there a relationship between women's climate change awareness levels and their fertility desire?
2. What is the relationship between women's climate change awareness levels and their fertility desire?

## 2 Materials and Methods

### 2.1 Type of Research

This study was conducted using a descriptive and correlational design and adhered to the STROBE guidelines for reporting (Von Elm et al. 2007).

### 2.2 Population and Sample of the Study

According to 2022 data from the Turkish Statistical Institute, the number of women aged 15–49 in Balıkesir was 312,541. Using a known population formula, with a 99% confidence interval and a 5% margin of error, the minimum required sample size was calculated as 384 women, using the OpenEpi program. The study was completed with a total of 440 women, and convenience sampling was utilized.

### 2.3 Setting

This research was conducted in a public hospital in western Türkiye.

### 2.4 Inclusion and Exclusion Criteria

Below are the characteristics of the women who were included in, and excluded from, the study.

Inclusion criteria:

- Aged 18–49 (reproductive age).
- Married.
- No children.

Exclusion criteria:

- Diagnosis of infertility.
- History of pregnancy loss.
- History of stillbirth.
- Loss of a child.
- Not proficient in Turkish.

### 2.5 Data Collection Forms Used in the Study

#### 2.5.1 Women's Demographic Characteristics Questionnaire

This form, developed by reviewing the literature, consists of 18 questions covering women's age, educational background, occupation, employment status, social security status, health status, marital status, and plans for having children (Arnocky et al. 2012; Schneider‐Mayerson and Leong 2020; Helm et al. 2021; Shahhosseini et al. 2024).

#### 2.5.2 Fertility Desire Scale

The 19‐item scale was developed by Naghibi et al. (2019) to measure the desire for having children among married individuals. The Turkish validity and reliability study of the scale was conducted by Kamiloğlu and Irmak Vural in 2022, with a Cronbach's alpha value of 0.821 (Kamiloğlu and Vural 2022). The scale consists of four sub‐dimensions: “Positive childbearing motivation,” “Preferences,” “Childbearing worries,” and “Social beliefs.” When calculating the scale score, each item is scored between 1 and 5. The lowest possible score from the entire scale is 19, and the highest possible score is 95. A higher scale score indicates more fertility desire. In both studies, the Cronbach's alpha value of the scale was found to be 0.82 (Naghibi et al. 2019; Kamiloğlu and Vural 2022). The Cronbach's alpha reliability coefficient for this study was found to be 0.66.

#### 2.5.3 Climate Change Awareness Scale

The Climate Change Awareness Scale was developed by Ataklı & Kuran, with its reliability and validity study conducted in 2022 (Ataklı and Kuran 2022). The scale, designed to assess climate change awareness, includes five sub‐dimensions: “Climate change awareness,” “Perception of the problem,” “Information on climate change causes,” “Climate change anxiety,” and “Behaviors and expectations from policies,” comprising a total of 52 items. It is based on a 5‐point Likert scale, with possible scores ranging from 52 to 260. Higher total and subscale scores indicate a greater level of climate change awareness. In the Turkish adaptation, Cronbach's alpha values for the overall scale and its subscales ranged from 0.80 to 0.93. In this study, the Cronbach's alpha value for the scale was found to be 0.81.

### 2.6 Data Collection Process

The questionnaires were administered through face‐to‐face interviews with women who met the research criteria and applied to the outpatient clinics of the obstetric and gynecological clinic between March and October 2023. Each participant completed the survey, which took an average of 15–20 min.

### 2.7 Ethical Considerations

Ethical approval was obtained from the Ethics Committee of Manisa Celal Bayar University Faculty of Medicine (Date: 2023; Number: 20478486‐739) and institutional permission was granted by Balıkesir Atatürk City Hospital (Date: 2023; Number: 220580412) before the study commenced. Permission to use the scales was secured via email from the developers. Additionally, written informed consent was obtained from participants through a voluntary consent form, in accordance with the principles of the Helsinki Declaration.

### 2.8 Data Analysis

Data were analyzed using SPSS (version 20.0). Descriptive statistics were used to examine the characteristics of the data, with continuous variables presented as mean ± standard deviation (SD), and categorical variables as frequencies and percentages (%). The normality of the data was assessed using kurtosis and skewness coefficients, with values within the range of ±2.0 considered acceptable (George and Mallery 2019). Differences between scale scores and independent variables were analyzed using the independent samples *t*‐test for two‐group comparisons and one‐way ANOVA for multiple group comparisons. The Bonferroni post‐hoc test was applied to identify which pairs of groups differed.

Multicollinearity among the explanatory variables was checked using the Variance Inflation Factor (VIF), and results showed no evidence of high collinearity (mean VIF = 0.017, maximum VIF = 0.18, and minimum VIF = 0.16). Multiple linear regression analysis was then conducted to determine the variables predicting the total score on the Fertility Desire Scale. Pearson's correlation was used to examine the relationship between Climate Change Awareness scores and Fertility Desire Scale scores. Statistical significance was set at *p* < 0.05.

## 3 Results

### 3.1 Sociodemographic Characteristics of Women

Table 1 presented the demographic characteristics of women and their relationship with fertility desire and climate change awareness scores. More than half of the women (55.2%) were aged 26 or younger. The majority (54.3%) had a university degree, and 34.8% were employed. Most participants had health insurance (93%), with 75.8% working in the labor sector. A large portion (89.1%) reported low or middle income. Most women (82.5%) resided in urban areas, and the vast majority (98.2%) lived in nuclear families. Additionally, 98.4% were in their first marriage, and 94.3% had couple‐initiated. The majority (83.4%) had been married for 0–2 years, with 95.5% planning to have children. Most women (86.4%) desired to have their first child within 1–3 years, and 58.4% preferred to have two children. A small proportion (7.5%) reported having a chronic disease.

**TABLE 1:** Demographic Characteristics and Their Relationship with Fertility Desire and Climate Change Awareness Scores (*n* = 440)

	Mean ± SD (min‐max)					
**Mean age at marriage (in months)**	26.2 ± 4.9 (17‐48)					
**Mean age of women (in years)**	24.4 ± 3.9 (18‐38)					
**Mean score of Fertility Desire Scale Total Score**	50.7 ± 7.6					
**Mean score of Climate Change Awareness Scale Total Score**	210.8 ± 23.2					

			Fertility Desire Scale Total Score		Climate Change Awareness Scale Total Score	
Characteristics	*n*	%	Mean ± SD	Test	Mean ± SD	Test
**Age**				t = −1.725		t = −3.265
≤ 26 years	243	55.2	50.1 ± 7.3	df = 438	207.5 ± 22.9	df = 418.432
> 26 years	197	44.8	51.4 ± 7.8	*p* = 0.085	214.7 ± 23.0	***p*** **= 0.001**
**Education level**						
Primary School (a)	37	8.4	56.8 ± 10.4	F = 14.356	188.4 ± 24.4	F = 29.191
High School (b)	164	37.3	50.5 ± 7.6	***p*** **= 0.000**	207.5 ± 20.6	***p*** **= 0.000**
University (c)	239	54.3	49.9 ± 6.6	**a > b.c**	216.4 ± 22.3	**a < b.c**
**Employment status**				t = −1.246		t = 2.931
Employed	153	34.8	50.1 ± 7.1	df = 438	215.2 ± 22.9	df = 313.492
Unemployed	287	65.2	51.0 ± 7.8	*p* = 0.213	208.4 ± 23.1	***p*** **= 0.004**
**Health insurance**				t = −2.196		t = 2.810
Yes	409	93.0	54.1 ± 9.2	df = 32.998	211.7 ± 22.7	df = 33.453
No	31	7.0	50.4 ± 7.4	***p*** **= 0.035**	198.0 ± 26.4	***p*** **= 0.008**
**Occupation**				t = 0.474		t = 1.093
Public Employee	37	24.2	50.5 ± 5.7	df = 151	218.7 ± 31.2	df = 151
Worker	116	75.8	49.9 ± 7.5	*p* = 0.636	214.0 ± 19.5	*p* = 276
**Perceived income level**				t = 2.312		t = −3.486
Low and middle income	392	89.1	50.4 ± 7.5	df = 58.393	209.4 ± 22.7	df = 57.577
High income	48	10.9	53.1 ± 7.7	***p*** **= 0.024**	222.2 ± 24.2	***p*** **= 0.001**
**Family type**						
Nuclear	432	98.2				
Extended	8	1.8	NA	NA	NA	NA
**Place of residence**						
City (a)	363	82.5	50.1 ± 7.1	F = 6.253	213.1 ± 21.5	F = 12.717
District (b)	41	9.3	53.7 ± 10.2	***p*** **= 0.002**	195.5 ± 28.3	***p*** **= 0.000**
Village (c)	36	8.2	53.1 ± 7.8	**b > a**	204.4 ± 26.8	**b < a**
**Marriage year**						
0‐2 year (a)	367	83.4	50.0 ± 7.2	F = 9.373	210.5 ± 21.3	F = 0.873
3‐5 year (b)	43	9.8	54.4 ± 8.5	***p*** **= 0.000**	209.5 ± 31.9	*P* = 0.418
≥5 year (c)	30	6.8	53.6 ± 9.1	b > a	216.1 ± 30.2	
**Type of marriage**				t = 2.902		t = −0.506
Arranged	25	5.7	55.9 ± 9.4	df = 25.793	208.4 ± 26.0	df = 438
Couple‐initiated	415	94.3	50.4 ± 7.3	***p*** **= 0.007**	210.9 ± 23.1	*p* = 0.613
**Number of marriages**						
First Marriag	433	98.4				
Second Marriage	7	1.6	NA	NA	NA	NA
**Planning to have children**				t = −0.075		t = 0.259
Yes	420	95.5	50.7 ± 7.5	df = 438	210.8 ± 22.9	df = 438
No	20	4.6	50.8 ± 8.7	*p* = 0.940	209.5 ± 30.8	*p* = 0.796
**Desired time to have a first child **				t = −0.314		t = 1.979
Within 1‐3 years	380	86.4	50.6 ± 7.3	df = 418	211.6 ± 22.6	df = 46.683
Within 4‐6 years	40	13.6	51.0 ± 9.4	*p* = 0.754	203.8 ± 23.9	*p* = 0.054
**Desired number of children**						
One child (a)	152	35.1	49.4 ± 6.5	F = 25.724	212.1 ± 23.0	
Two children (b)	253	58.4	50.5 ± 7.2	***p*** **= 0.000**	209.8 ± 22.5	F = 0.526
Three and more children (c)	28	6.5	59.9 ± 9.9	**c > a,b**	209.5 ± 28.6	*p* = 0.591
**Condition of chronic disease**				t = 2.257		t = 0.918
Yes	33	7.5	54.9 ± 11.4	df = 34.042	214.3 ± 32.7	df = 438
No	407	92.5	50.3 ± 7.1	***p*** **= 0.031**	210.5 ± 22.3	*p* = 0.359

*Note*: A *p* value of <0.016 was considered significant according to the Bonferroni post‐hoc test, 10 low‐income individuals were merged with the medium‐income group.

Bold values indicate statistically significant results. A *p*‐value of <0.016 was considered significant for comparisons using the Bonferroni post‐hoc test; for other analyses, *p* <0.05 was considered significant.

### 3.2 Comparison of the Descriptive Characteristics and the Fertility Desire Scale Total Score

The mean total score of the Fertility Desire Scale was 50.7 ± 7.6. The mean Fertility Desire Scale score differed significantly based on education level (*p* = 0.000), with primary school graduates scoring higher (56.8 ± 10.4) compared to high school (50.5 ± 7.6) and university graduates (49.9 ± 6.6). Participants with health insurance and high incomes had higher scores compared to those without health insurance and with low or middle incomes. Participants living in districts scored higher (53.7 ± 10.2) than those living in cities (50.1 ± 7.1). Women married for 3–5 years (54.4 ± 8.5) scored higher compared to those married for less than 2 years (50.0 ± 7.2). Women in arranged marriages had significantly higher scores compared to those in couple‐initiated marriages (*p* = 0.007). The average score of women who had been desiring children for 3 years or more was higher than that of others (*p* < 0.05). Individuals with chronic illnesses were found to have a higher fertility desire. Fertility Desire Scale scores were similar across age, employment status, occupation, planning to have children, and desired time to have a first child (Table 1).

### 3.3 Comparison of the Descriptive Characteristics and Climate Change Awareness Scale Total Score

The Climate Change Awareness Scale had a mean total score of 210.8 ± 23.2. Those over the age of 26, who were employed, had social security, and a high income level demonstrated higher awareness of climate change compared to others (*p* < 0.05). The average score of participants with primary school education (188.4 ± 24.4) was lower than those with high school (207.5 ± 20.6) and university degrees (216.4 ± 22.3). Scale scores for individuals living in districts (195.5 ± 28.3) were lower compared to those living in cities (213.1 ± 21.5). No significant differences were observed in climate change awareness scores based on occupation, years of marriage, type of marriage, planning to have children, desired timing for the first child, or having a chronic disease (Table 1).

### 3.4 Variables That Predict the Total Score of the Fertility Desire Scale Total Score

A multiple linear regression analysis was conducted to predict the desire to have children based on variables including education level, health insurance, place of residence, years of marriage, type of marriage, perceived income level, and climate change awareness. The analysis results showed that the model was statistically significant, *F* (10,409) = 7.822, *p* < 0.001. Among the variables in the model, all were statistically significant predictors of the desire to have children, except for health insurance and place of residence (*p* < 0.05). The model explained 14.0% of the variance in Fertility Desire. Those who had been married for 3–5 years and those married for 5 years or more had a higher desire to have children compared to those who had been married for 2 years or less.

Individuals in arranged marriages exhibited a higher fertility desire compared to those in couple‐initiated marriages. Fertility desire was higher among individuals with a high income level compared to those with low or middle income levels. Women with higher climate change awareness exhibited a lower fertility desire compared to those with lower awareness (Table 2).

**TABLE 2:** Variables that predict the total score of the Fertility Desire Scale Total Score.

	Unstandardized coefficients		Standardized coefficients			95% CI for *B*	
	*β*	SD	Beta	*t*	*p*	Lower bound	Upper bound
Constant	64.597	3.255		19.843	0.000^*^	58.197	70.996
Education (high school)	−0.991	1.425	−0.074	−0.696	0.487	−3.792	1.810
Education (university)	−1.496	1.485	−0.115	−1.007	0.314	−4.414	1.423
Marriage year (3–5 years)	3.236	1.043	0.144	3.103	0.002^*^	1.186	5.286
Marriage year (≥5 years)	3.466	1.262	0.134	2.747	0.006^*^	0.985	5.947
Type of marriage (arranged)	−3.890	1.430	−0.132	−2.720	0.007^*^	−6.701	−1.079
Health insurance (yes)	1.079	1.263	0.042	0.854	0.393	−1.403	3.561
Perceived income level (high income)	4.203	0.971	0.204	4.327	0.000^*^	2.294	6.113
Place of residence (district)	1.550	1.438	0.069	1.078	0.282	−1.277	4.377
Place of residence (city)	−0.661	1.194	−0.039	−0.554	0.580	−3.009	1.686
Climate Change Awareness Scale Total Score	−0.048	0.014	−0.172	−3.455	0.001^*^	−0.076	−0.021

* *p* < 0.05; *R* ^2^ = 0.16; Adj*R* ^2^ = 0.14.

According to the regression analysis results, the regression equation predicting Fertility Desire is as follows: Fertility Desire = (−0.053 × Climate Change Awareness) + (64.964). An increase of 1 unit in the Climate Change Awareness score corresponds to a decrease of 0.053 units in Fertility Desire. The results are presented in Table 2, and Climate Change Awareness alone explains 3.1% of the variance in Fertility Desire (Table 3).

**TABLE 3:** Climate change awareness that predict the total score of the Fertility Desire Scale Total Score.

	Unstandardized coefficients		Standardized coefficients			95% CI for *B*	
	*β*	SD	Beta	*t*	*p*	Lower bound	Upper bound
Constant	60.650	2.879		21.068	0.000^*^	21.068	0.000
Climate Change Awareness Scale Total Score	−0.050	0.014	−0.177	−3.675	0.000^*^	−3.675	0.000

* *p* < 0.05; *R* ^2^ = 0.031; Adj*R* ^2^ = 0.029.

### 3.5 Correlation Between Fertility Desire Scale and Climate Change Awareness Scale

**FIGURE 1:**
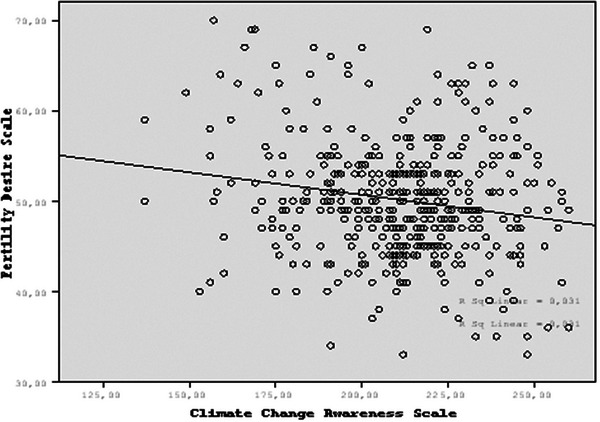
Scatterplot of the scales.

Fertility desire was weakly negatively correlated with information on climate change causes (*r* = −0.188), climate change anxiety (*r* = −0.187), behaviors and expectations from policies (*r* = −0.141), and overall climate change awareness (*r* = −0.151) (*p* < 0.01) (Table 4). The scatterplot illustrates the relationship between the Fertility Desire Scale and the Climate Change Awareness Scale. A weak negative correlation is evident, as indicated by the slight downward slope of the trend line (Figure 1).

**TABLE 4:** Correlation between the total and subscales of the Fertility Desire Scale and the Climate Change Awareness Scale.

		Climate change awareness	Perception of the problem	Information on climate change causes	Climate change anxiety	Behaviors and expectations from policies	Climate Change Awareness Scale
Positive childbearing motivation	*r*	−0.141	−0.019	−0.175	−0.212	−0.142	−0.186
	*p*	**0.003**	0.698	**0.000**	**0.000**	**0.003**	**0.000**
	*n*	440	440	440	440	440	440
Preferences	*r*	0.101	0.072	0.074	0.017	0.043	0.074
	*p*	**0.035**	0.130	0.123	0.720	0.367	0.123
	*n*	440	440	440	440	440	440
Childbearing worries	*r*	0.081	−0.052	−0.009	−0.029	−0.026	−0.009
	*p*	0.090	0.272	0.843	0.539	0.582	0.854
	*n*	440	440	440	440	440	440
Social beliefs	*r*	0.083	−0.041	−0.209	−0.078	−0.109	−0.093
	*p*	0.082	0.389	**0.000**	0.100	**0.022**	**0.050**
	*n*	440	440	440	440	440	440
Fertility Desire Scale	*r*	−0.008	−0.028	−0.188	−0.187	−0.141	−0.151
	*p*	0.873	0.559	**0.000**	0.**000**	**0.003**	**0.002**
	*n*	440	440	440	440	440	440

*Note*: Bolded values are *p* < 0.05.

## 4 Discussion

In this study, the awareness levels of women regarding climate change and its impact on their fertility desire were examined. It was found that women assessed their desire for fertility in their future lives, with the vast majority (95.5%) expressing a desire to have children. More than half of the participants were university graduates. The average age of the participants was 24, and most had been married for 2 years or less. While climate change awareness was above average, fertility desire was at an average level.

In previous studies conducted with women who had given birth (Başaran and Çetik 2024) and with fertile and infertile individuals (Shahhosseini et al. 2024), the average fertility desire scores were reported as 67.95 ± 11.85 and 60.82 ± 9.36, respectively. In our study, however, the average fertility desire score was found to be 50.7 ± 7.6. This difference may be due to the diversity of demographic and clinical characteristics in the study groups. According to the results of the regression analysis, individuals who had been married for 3–5 years or more than 5 years had a higher fertility desire compared to those who had been married for 0–2 years. Unlike our study findings, other studies did not find a relationship between fertility desire and years of marriage (Arasteh et al. 2022; Ghaffari et al. 2024). However, there was a study that found a negative relationship between years of marriage and fertility desire (Shahhosseini et al. 2024). In Turkish society, it is believed that in long‐term marriages, factors such as couples feeling increased pressure to have children or approaching the biological limits of childbearing age affect fertility desire. These differing results between studies can be explained by the influence of different sample groups and cultural contexts.

Similar to our study, the literature also indicated that fertility was lower in couple‐initiated marriages compared to arranged marriages. The reason for this was thought to be that in marriages chosen by individuals themselves, the focus of the marriage and sexual relationship shifted from extended family responsibilities to the couple's personal needs, which in turn reduced fertility (Lesthaeghe 2010; Manglos‐Weber and Weinreb 2017).

In this study, women who reported having a high income were found to have had a greater desire for fertility compared to those with low and middle income. Other studies also showed that individuals with higher socioeconomic status generally had a stronger desire to have more children (Saya et al. 2021; Shahhosseini et al. 2024). However, a study indicated that, in developed countries, rather than income levels, fertility desire was negatively influenced by the fact that an increase in the number of children would raise the carbon footprint, thereby contributing to climate change (Schneider‐Mayerson and Leong 2020). Considering the economic burden of having children, the findings of this study were consistent with the literature.

The multiple regression analysis showed that climate change awareness reduces fertility desire, a finding supported by other studies. Schneider‐Mayerson and Leong (2020) found that a significant proportion of young adults in the United States consider climate change when making reproductive choices, with 59.8% expressing concern about the carbon footprint of having children (Schneider‐Mayerson and Leong 2020). Similarly, Arnocky et al. (2012) found that environmental concerns were associated with lower fertility intentions among Canadian students (Arnocky et al. 2012). Helm et al. (2021) reported that individuals in New Zealand and the United States chose to remain childfree due to climate‐related concerns such as overpopulation and resource scarcity (Helm et al. 2021). These countries, as examples of developed nations, demonstrate how high environmental awareness significantly influences fertility preferences. On the other hand, Haq (2018) examined the impact of environmental disasters, such as floods, on fertility decisions in rural Bangladesh, finding that families viewed children as a form of security against environmental challenges (Haq 2018). These findings indicate that in developing countries like Bangladesh and Sub‐Saharan Africa, environmental factors have an indirect impact on fertility decisions. Türkiye's classification as a developing country and the fact that the study was conducted in the western region, which has a higher socio‐economic development level according to the Socio‐Economic Development Ranking Survey 2017 (General Directorate of Development Agencies 2019), support the indirect impact of environmental factors on fertility decisions. This trend resembles the effect of environmental concerns on fertility preferences in developed countries. Consequently, while climate change awareness reduces the desire to have children in developed countries, this effect is beginning to be felt in developing countries with the rise of environmental awareness. In less developed countries, however, the decision to have children is mainly driven by economic needs, with lower levels of environmental awareness (Haq 2018; Bongaarts 2020). In this context, there are significant differences in the relationship between climate change awareness and fertility decisions based on the level of development of the countries. However, in disaster‐prone areas like rural Bangladesh, natural events like floods may increase fertility desire for economic reasons, contrasting with the long‐term existential concerns seen in developed countries. These findings highlight how climate change awareness consistently reduces fertility desires in developed regions, while socio‐economic and immediate risks may have the opposite effect in vulnerable areas.

The findings indicated a weak negative correlation between fertility desire and various aspects of climate change awareness. Negative correlation coefficients for information about climate change causes, climate change anxiety, behaviors and policy expectations, and overall climate change awareness suggested that as awareness or concern about climate change increased, fertility desire slightly decreased. The slight downward slope of the trend line in the scatterplot also confirmed this weak relationship, demonstrating that individuals with higher levels of climate change awareness or anxiety tended to have a somewhat reduced desire to have children. However, the correlation was weak, indicating that climate change awareness was not a significant determinant of fertility desire. The lack of a strong relationship between women' desire to have children and their concerns about climate change can be explained by the fact that the decision to have children is influenced by many different factors. This suggests that fertility desire is shaped not only by climate concerns but also by economic conditions, social pressures, personal values, and various other factors. Dillarstone et al. (2023) also found a complex relationship between climate change concerns and reproductive decisions based on ethical, environmental, livelihood and political considerations. In conclusion, given the numerous factors that can influence reproductive desire, there is a need for further research and studies conducted with larger populations in this area.

## 5 Conclusion

In conclusion, this study highlights the multifaceted nature of factors influencing fertility desire among women, particularly the interplay between climate change awareness and reproductive intentions. While climate change awareness was found to have a weak negative correlation with fertility desire, it was not a dominant determinant. The findings align with previous research, which indicates that while climate change concerns do affect reproductive attitudes, they coexist with a range of other influential factors such as socio‐economic status, marital duration, and cultural expectations. The results underscore that in developing countries like Türkiye, with regions exhibiting higher socio‐economic development, environmental concerns are beginning to impact fertility decisions similarly to patterns observed in developed nations. However, in less developed areas, economic and immediate survival needs remain the primary drivers of fertility desire. Future research involving larger, more diverse populations is essential to deepen the understanding of how environmental awareness and other variables interact to shape reproductive choices in different socio‐economic contexts. This would provide a clearer insight into the evolving dynamics of fertility desire as environmental awareness continues to grow globally.

### 5.1 Strengths and Limitations

#### 5.1.1 Strengths

One of the strengths of the study is that it was conducted with women of reproductive age who did not have any health issues preventing them from having children or affecting their fertility. This contributed to the homogeneity of the results and their reflection of a specific population.

#### 5.1.2 Limitations

Due to the sampling methodology used in this study, the results cannot be considered as generalizable. However, the low Cronbach's alpha value of the fertility desire scale, being below 0.7, has limited the reliability of the study. The inclusion of items such as “Preventing pregnancy is interfering with God's will” in the scale may have impacted the results. Such questions with religious or cultural connotations could influence participants' responses and reduce the overall internal consistency of the scale. Participants may provide different or inconsistent answers due to personal, religious, or cultural sensitivities, which could lead to a lower Cronbach's alpha value and limit the reliability of the scale.

## Ethics Statement

Ethical approval for this study was granted by the Ethics Committee of Manisa Celal Bayar University Faculty of Medicine (Approval Number: 20478486‐739; Date: 2023). Authorization to use the scales was obtained through email correspondence with their original developers.

## Consent

Participants provided written informed consent through a voluntary consent form, adhering to the principles outlined in the Helsinki Declaration.

## Conflicts of Interest

The authors declare no conflicts of interest.

## Data Availability

The data presented in this study are available upon request from the corresponding author.
